# Binaural advantages in sound temporal information processing by neurons in the rat inferior colliculus

**DOI:** 10.3389/fnins.2023.1308052

**Published:** 2023-12-06

**Authors:** Yan Li, Jiping Zhang

**Affiliations:** Key Laboratory of Brain Functional Genomics, Ministry of Education, NYU-ECNU Institute of Brain and Cognitive Science at NYU Shanghai, School of Life Sciences, East China Normal University, Shanghai, China

**Keywords:** gap detection threshold, temporal information processing, binaural advantage, inferior colliculus, rats

## Abstract

Previous studies on the advantages of binaural hearing have long been focused on sound localization and spatial stream segregation. The binaural advantages have also been observed in speech perception in reverberation. Both human speech and animal vocalizations contain temporal features that are critical for speech perception and animal communication. However, whether there are binaural advantages for sound temporal information processing in the central auditory system has not been elucidated. Gap detection threshold (GDT), the ability to detect the shortest silent interval in a sound, has been widely used to measure the auditory temporal resolution. In the present study, we determined GDTs of rat inferior collicular neurons under both monaural and binaural hearing conditions. We found that the majority of the inferior collicular neurons in adult rats exhibited binaural advantages in gap detection, i.e., better neural gap detection ability in binaural hearing conditions compared to monaural hearing condition. However, this binaural advantage in sound temporal information processing was not significant in the inferior collicular neurons of P14-21 and P22-30 rats. Additionally, we also observed age-related changes in neural temporal acuity in the rat inferior colliculus. These results demonstrate a new advantage of binaural hearing (i.e., binaural advantage in temporal processing) in the central auditory system in addition to sound localization and spatial stream segregation.

## 1 Introduction

Binaural listeners show remarkable abilities in sound localization by utilizing interaural time and level difference cues, as well as spectral cues (Grothe et al., 2010; Keating and King, 2015). Previous studies have shown that human subjects localize sound sources more accurately in binaural listening conditions than in monaural listening conditions, demonstrating a binaural advantage in sound localization (Butler, 1994; Van Wanrooij and Van Opstal, 2007). Unilateral hearing loss impairs horizontal sound localization accuracy (Parisa et al., 2017), and disrupts normal neural tuning to sound-source azimuth of auditory cortical neurons (Wang et al., 2019).

Apart from the binaural advantage in sound localization, binaural listening benefit was also observed in speech perception in noisy and reverberant environments (Dubno et al., 2008). Studies on speech perception have shown the binaural advantages on speech intelligibility and response time under binaural vs. monaural listening conditions (Yancey et al., 2021). The speech discrimination is better binaurally than monaurally under reverberation for both normally hearing and hearing-impaired subjects (Gelfand and Hochberg, 1976). Besides, unilateral hearing loss impairs the recognition of speech in competing speech (Siegbahn et al., 2021), and the adult patients with bilateral cochlear implants demonstrated a bilateral advantage for speech perception in bilateral listening mode compared to unilateral listening mode in both quiet and noisy environments (Litovsky et al., 2006; Mosnier et al., 2009). Speech perception in noise is shown to be improved after corrected congenital unilateral conductive hearing loss (Wilmington et al., 1994). In addition, binaural hearing also improves the ability to segregate target signals from noise or competing source in real-world auditory scenes (Avan et al., 2015; Middlebrooks and Waters, 2020). Therefore, binaural hearing plays critical roles in sound localization, stream segregation, and speech perception in noisy and reverberant environments.

Human speech and animal communication sounds contain rapidly changing temporal information. The ability of the auditory system to resolve sound temporal information is crucial for perceiving speech, communication sounds, and other complex auditory stimuli (Snell et al., 2002; Babkoff and Fostick, 2017). However, there are few studies on the binaural advantages in sound temporal information processing. Gap detection threshold (GDT), which measures the ability to detect the shortest silent interval in a sound, is widely used to assess the auditory temporal resolution and evaluate the ability of auditory system in sound temporal processing (Werner et al., 1992; Pysanenko et al., 2021; Land and Kral, 2022). Shorter GDTs indicate higher temporal acuity in decoding of the sound stream, such as speech stream of human and communication sounds of animals. In contrast, longer than normal GDTs may result in slower auditory processing speed and difficulty in decoding sound streams. Studies on young children (Yuvaraj et al., 2023) and rodents (Karcz et al., 2015) have demonstrated binaural advantages in gap detection, i.e., a better performance in gap detection task in binaural condition compared to monaural condition. However, the neural mechanism underlying the binaural advantage in gap detection ability is not understood.

The neuronal correlates of gap detection in the central auditory system have been determined in the inferior colliculus (Walton et al., 1997; Weible et al., 2020; Land and Kral, 2022), auditory thalamus (Anderson and Linden, 2016), and auditory cortex (Eggermont, 1999; Recanzone et al., 2011; Jiang et al., 2015; Zhao et al., 2015; Keller et al., 2018; Awwad et al., 2020) across different animal species. These studies determined the GDTs of auditory neurons under either binaural conditions or monaural conditions without a direct comparison of GDTs under both conditions. A direct comparison of the GDTs of central auditory neurons determined under binaural versus monaural conditions can help us to understand whether there are any binaural advantages in temporal information processing at the neuronal level.

The inferior colliculus is an important center for processing auditory information in the central auditory system. It receives convergent inputs from a large number of lower auditory nuclei, e.g., the ascending inputs from cochlear nucleus, the superior olive complex, and lateral leminiscus (Kelly et al., 1998; Frisina, 2001). It also receives descending feedback modulation from auditory cortex (Herbert et al., 1991; Blackwell et al., 2020; Qi et al., 2020) and medial geniculate body (Winer et al., 2002; Wu and Yan, 2007). The inferior collicular neurons integrate binaural information from both ears (Irvine and Gago, 1990; Zhang and Kelly, 2009). The purpose of this study is to determine whether there are any binaural advantages in the gap detection ability of neurons in the rat inferior colliculus. We measured the GDTs of inferior collicular neurons in rats under both binaural and monaural stimulus conditions. We found that the majority of inferior collicular neurons in adult rats exhibited a binaural advantage in gap detection, i.e., GDTs were lower under binaural hearing conditions than under monaural hearing conditions. However, we did not find this binaural advantage from the population of neurons in the inferior colliculus in the postnatal day (P) 14–21 rats and the P22-30 rats.

## 2 Materials and methods

### 2.1 Animals and surgery

Three age groups of Sprague–Dawley rats were used in the present study: (1) P14-21 group, consisting of P14-21 rats (*n* = 82); (2) P22-30 group, consisting of P22-30 rats (*n* = 71); (3) adult group, consisting of P57-70 rats (*n* = 66). These rats were obtained from in-housing breed stocks that originated from the breeding pairs purchased from Shanghai Jie Si Jie Laboratory Animal Co., Ltd. (Shanghai, China). The immature rats were raised with their parents until reaching P26. All rats were reared in the housing environments (20–24°C room temperatures) with 12 h light/dark cycles, and had free access to water and food.

The rats were anaesthetized with urethane (i.p., 1.5 g/kg body weight) before surgery, and the anesthesia was maintained during the experiment through additional injections when necessary. The trachea was cannulated, and atropine sulfate (0.01 mg/kg, s.c.) was given to the rat subcutaneously to reduce bronchial secretions. The rat’s body temperature was monitored and maintained at 37.5°C using a feedback-controlled heating blanket. The dorsal skull was exposed, and a nail (4 cm long) was attached to the frontal dorsal surface of the skull with 502 super glue and dental cement. The head of the rat was then fixed by the nail to a head holder attached to a stainless steel platform. For the adult rats, a craniotomy was performed over the left cortex above the inferior colliculus (AP: 8.1 to 9.1 mm posterior to Bregma; ML: 1.2 to 2.2 mm from midsagital reference to lateral) based on stereotaxic coordinates of the rat brain (Paxinos and Watson, 2007). For the immature rats, the position for the craniotomy was modified based on the distances between Bregma and Lambda measured from these rats, and the atlas of the postnatal rat brain in stereotaxic coordinates (Khazipov et al., 2015). A small incision was made in the dura to expose a portion of cortex for inserting electrodes. Warm saline was applied onto the exposed brain surface during the experiment to prevent drying.

### 2.2 Acoustic stimuli

Acoustic stimuli were presented through a PC-controlled an auditory neurophysiology workstation (Tucker-Davis Technologies, USA). The hardware of the workstation for presenting acoustic stimuli included a multifunction processor (RX6-A5), a stereo power amplifier (SA1), and two multi-field magnetic speakers (MF1). The acoustic stimuli were delivered to the ears of the rats via a close-field system similar to our previous studies (Liu et al., 2021). Briefly, the MF1 speakers were incorporated internal parabolic cones and coupled to the ears through PVC plastic tubes (9.5 cm long, 1/16 inch ID, 1/8 inch OD, and 1/32 inch wall thickness) leading to the ear canals. Adaptable plastic tubes were used when necessary to couple the ear canals of infant rats. The distance from the end of the tube in the ear canal to the tympanic membrane was approximately 5 mm. The output of each MF1 speaker was calibrated from 2.0 to 44.0 kHz (sampling rate, 100 kHz) using a 1/4 inch condenser microphone (model 7,016, ACO Pacific Inc.) coupled to the end of the plastic tube (with 5 mm distance to the microphone) with a suitable latex tube. The calibration data from the MF1 speakers were stored in computer and used for obtaining the desired sound pressure levels in decibel (dB SPLs, re: 20 μPa) within frequency ranging from 2.0 to 44.0 kHz. This close-field sound delivery system was used to precisely control the acoustic stimulus parameters presented in the two ears.

The acoustic stimuli were either pure tones (100 ms duration, 5 ms rise-fall time) or noise bursts (broadband white noise, 4.0–44.0 kHz, 100 ms duration) shaped with linear rise and fall functions (1 ms rise time and 1 ms fall time). The tone bursts were used to search the characteristic frequency (*CF*) of the inferior collicular neurons, and the noise bursts were used to measure the GDTs of rat inferior collicular neurons. The acoustic stimuli were presented monaurally to either ear, or binaurally to both ears simultaneously.

Gap stimuli (i.e., pairs of noise bursts with varying durations of silent gaps) were used to measure the GDT of each neuron. The two noise bursts were identical and separated by a silent gap between them. The gap durations were varied from 0 ms to 200 ms (i.e., 0, 2, 4, 6, 8, 10, 15, 20, 30, 40, 50, 100, 150, and 200 ms). Under the condition of the 0 ms gap duration, the downward ramp of the leading noise burst (noise burst 1, NB1) and the upward ramp of the lagging noise burst (noise burst 2, NB2) abutted each other without a true silent gap between NB1 and NB2. To create a gap between NB1 and NB2, the onset of NB2 related to the recording epoch was held constant at 400 ms, whereas the silent interval (gap duration) between NB1 and NB2 was varied. The inter-trial interval for successive stimulus trials of stimuli was 1,200 ms.

### 2.3 Recording system

The responses of rat inferior collicular neurons to acoustic stimuli were recorded in a sound-insulated double-walled room. Glass electrodes (1.0–2.0 MΩ impedance, filled with 2 M NaCl) were advanced from the cortex to the inferior colliculus at an angle of 10° from the frontal plane using a remote controlled microdrive (SM-21, Narishige, Japan). The signal from the electrode was amplified (1000×) and filtered (0.3–3.0 kHz) by a DAM80 pre-amplifier (WPI, United States), digitized by a RZ-5 Bioamp data processor (TDT3, United States), and then stored in the computer for both online and off-line analyses. Simultaneously, the electrode signal was also sent to a digital oscilloscope (TDS 2024, USA) and an audio speaker for online monitoring.

### 2.4 Data collection and analysis

In the present study, we focused on recording in the central nucleus of the inferior colliculus (ICC). For each rat, preliminary electrode penetrations were made in the ICC to search for a tonotopic trend, i.e., an increase in characteristic frequency (*CF*) along the dorsal to ventral direction. The *CF* was defined as the tonal stimulus frequency at which the neuron had the lowest response threshold. For each rat, the ICC was localized based on the stereotaxic coordinates (Paxinos and Watson, 2007; Khazipov et al., 2015), the distance between the bregma and lambda of each rat, and the physiological criteria such as a low-to-high gradient of *CF* from dorsal-to-ventral direction, short-latency responses to *CF* stimuli, and sharp frequency tuning (Langner et al., 2002; Malmierca et al., 2008; Yao et al., 2015). To search the tonotopy, we determine the CFs of these neurons audio-visually. Once the ICC was identified based on recorded neuronal response properties from the preliminary electrode penetrations, subsequent single-unit recording was performed along the tonotopic axis. To determine the GDTs of ICC neurons, we only collected data from ICC neurons with onset response properties. Once an ICC neuron was well isolated, the *CF* of the neuron was determined by using a frequency-level matrix. The tonal stimulus levels were equal in both ears ranging from 80 to 10 dB SPL with decrements of 10 dB steps. The frequencies were varied from 2 kHz to 44 kHz with a step size of 1 kHz.

The rate-level functions of the neuron responding to monaural stimuli in either ear were obtained using noise stimuli. The noise levels were varied from 0 to 80 dB SPL in 10-dB steps. The ICC neurons of rats were classified as EE, EO, or OE based on their monaural responses and a previous classification scheme (Zhang et al., 2004). A neuron was categorized as EO if it responded exclusively to monaural stimulation in the contralateral ear but not in the ipsilateral ear; as EE if it responded to monaural stimulation of either ear; and as OE if the neuron responded to monaural stimulation in the ipsilateral ear but not in the contralateral ear. For the neurons we recorded, only a few neurons were OE; therefore, we focused solely on the analying neuronal responses to the gap stimuli in both EO and EE neurons. The dominant ear was defined as the ear that exhibited greater responses to the same acoustic stimuli in the rate-level functions. Our data showed that the dominant ear was the contralateral ear for all EO neurons and for most of the EE neurons. The binaural interaction type of a neuron was determined under each binaural condition that was used to measure the GDT. The protocol to determine the binaural interaction type was as follows: the responses of the neuron to a 60 dB SPL monaural noise stimulus in the dominant ear was determined, and the responses of the neuron to binaural stimuli were then determined by fixing the noise level in the dominant ear at 60 dB SPL whereas the noise levels in the non-dominant ear were varied at 40, 50, 60, 70, and 80 dB SPL, respectively. Each stimulus condition was repeated 30 times. The binaural interaction type of a neuron under a specific binaural stimulus condition was categorized as facilitatory if its binaural response were 20% greater than the sum of the monaural responses, inhibitory if its responses under a binaural condition were 20% smaller than the sum of monaural responses, and no interaction if the difference between its binaural response and the sum of the monaural responses fell within −20 to 20%.

**Figure 1:**
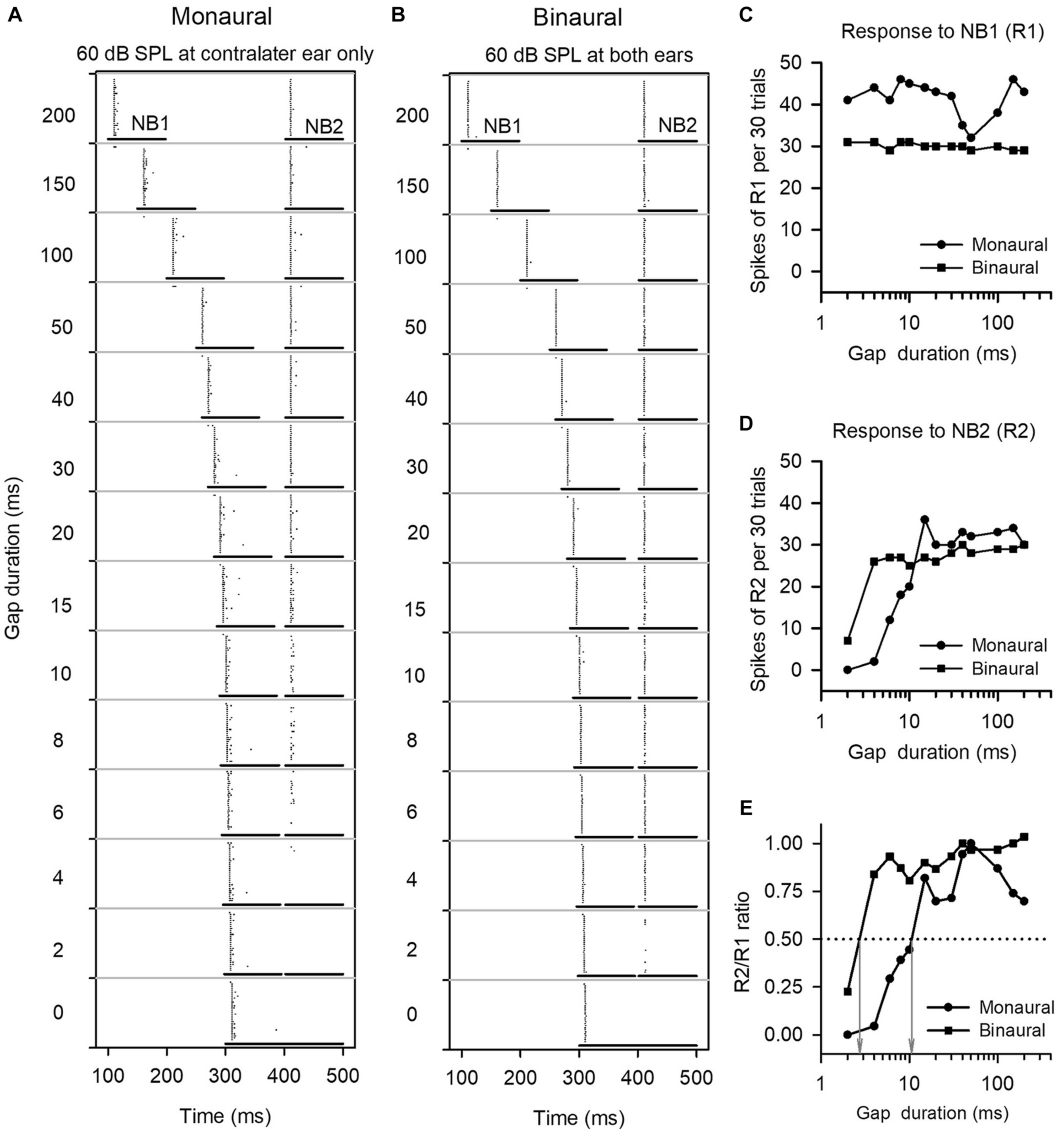
The method to determine the gap detection threshold (GDT) of a neuron. **(A,B)** Dot-raster representations of an ICC neuron responding to gap stimuli at various gap durations under monaural and binaural stimulus conditions. Each dot is an action potential. The two horizontal lines represent the noise burst 1 (NB1) and noise burst 2 (NB2), and the silence gap durations (ms) between NB1 and NB2 are shown on the left of each panel. At each gap duration condition, the gap stimuli were repeated 30 times. **(C,D)** Line drawings of the responses to NB1 (R1) and the responses to NB2 (R2). **(E)** R2/R1 ratio. The gap duration at which the R2/R1 ratio equals 0.5 is defined as the GDT of the neuron (see gray arrows). For this neuron, the GDTs are 10.7 ms under monaural stimulus condition, and 2.7 ms under binaural stimulus condition, respectively.

To determine the GDT of a neuron under monaural conditions, the noise level in the dominant ear was fixed at 60 dB SPL and no stimuli were presented to the other ear. To determine the GDT of a neuron at binaural conditions, the noise level in the dominant ear was fixed at 60 dB SPL, while varying the noise levels in the other ear (40, 50, 60, 70, and 80 dB SPL respectively). For convenience to present the population data in results, the binaural stimulus conditions used to determine the binaural GDTs can also be defined in terms of the interaural level differences (ILDs, the sound level in the dominant ear minus the sound level in the other ear), such as ILD +20, ILD +10, ILD 0, ILD −10, and ILD −20 in dB, respectively. These ILD values are only used to indicate different binaural stimulus conditions and not spatial positions. At each monaural or binaural stimulus condition, the responses of ICC neurons to the pairs of noise stimuli (NB1 and NB2) at the designed gap durations were recorded. The responses were determined as the total number of spikes collected within each noise burst duration for 30 trials of stimuli. A response ratio was calculated by dividing the response to NB2 by the response to NB1, and then the response ratio vs. gap duration function was plotted for each neuron. The neuronal GDT under monaural or binaural stimulus conditions was defined as the gap duration corresponding to a ratio of 0.5 in the response ratio vs. gap duration function (Figure 1).

Statistical analysis was performed on the population data using the Kruskal-Wallis test and Mann–Whitney test for nonparametric independent samples, as well as the Friedman test and Wilcoxon Signed Rank test for nonparametric related samples, with a significance level at 0.05.

## 3 Results

The data shown in the present study are from 399 neurons in the ICC of three age groups of rats, including 136 neurons in the adult group, 129 neurons in the P14-21 group, and 134 neurons in the P22-30 group. We determined the responses of the ICC neurons to gap stimuli under both monaural and the five binaural conditions (i.e., ILD +20 dB, ILD +10 dB, ILD 0 dB, ILD −10 dB, and ILD −20 dB, respectively). For some neurons, we failed to record the responses to gap stimuli under certain binaural conditions (e.g., ILD −20 dB and ILD −10 dB) due to strong binaural inhibition. Additionally, due to time consuming nature of data collection, we also encountered instances where we failed to collect the data under some of the five binaural conditions for certain neurons. Consequentially, in the population data analysis, the total numbers of neurons with the binaural vs. monaural data pairs are not equal at the tested five binaural conditions.

### 3.1 The responses of ICC neurons to gap stimuli under both monaural and binaural conditions

We determined the responses of each ICC neuron to gap stimuli with varying gap durations, and then compared the GDTs of each neuron measured under the monaural vs. binaural hearing conditions. Figure 1 shows the responses of an ICC neuron to gap stimuli under both monaural (60 dB SPL at contralateral ear only) and binaural (60 dB SPL at both ears) hearing conditions. This neuron responded relatively stable to noise burst 1 (NB1) under both the monaural and the binaural stimulus conditions (Figures 1A–D). Under the monaural stimulus conditions, the neuron exhibited weak responses to noise burst 2 (NB2) when the gap duration was 6 ms, and showed almost no responses to NB2 when gap durations were between 0 ms and 4 ms (Figure 1A); in contrast, under the binaural stimulus conditions, the neuron exhibited a relative strong response to NB2 when the gap duration was 4 ms (Figure 1B). The GDTs of this neuron were 10.7 ms in the monaural hearing condition and 2.9 ms in the binaural hearing condition (Figure 1E). The data indicate a binaural advantage in gap detection, i.e., a better gap detection ability in the binaural hearing condition compared to the monaural hearing condition.

**Figure 2:**
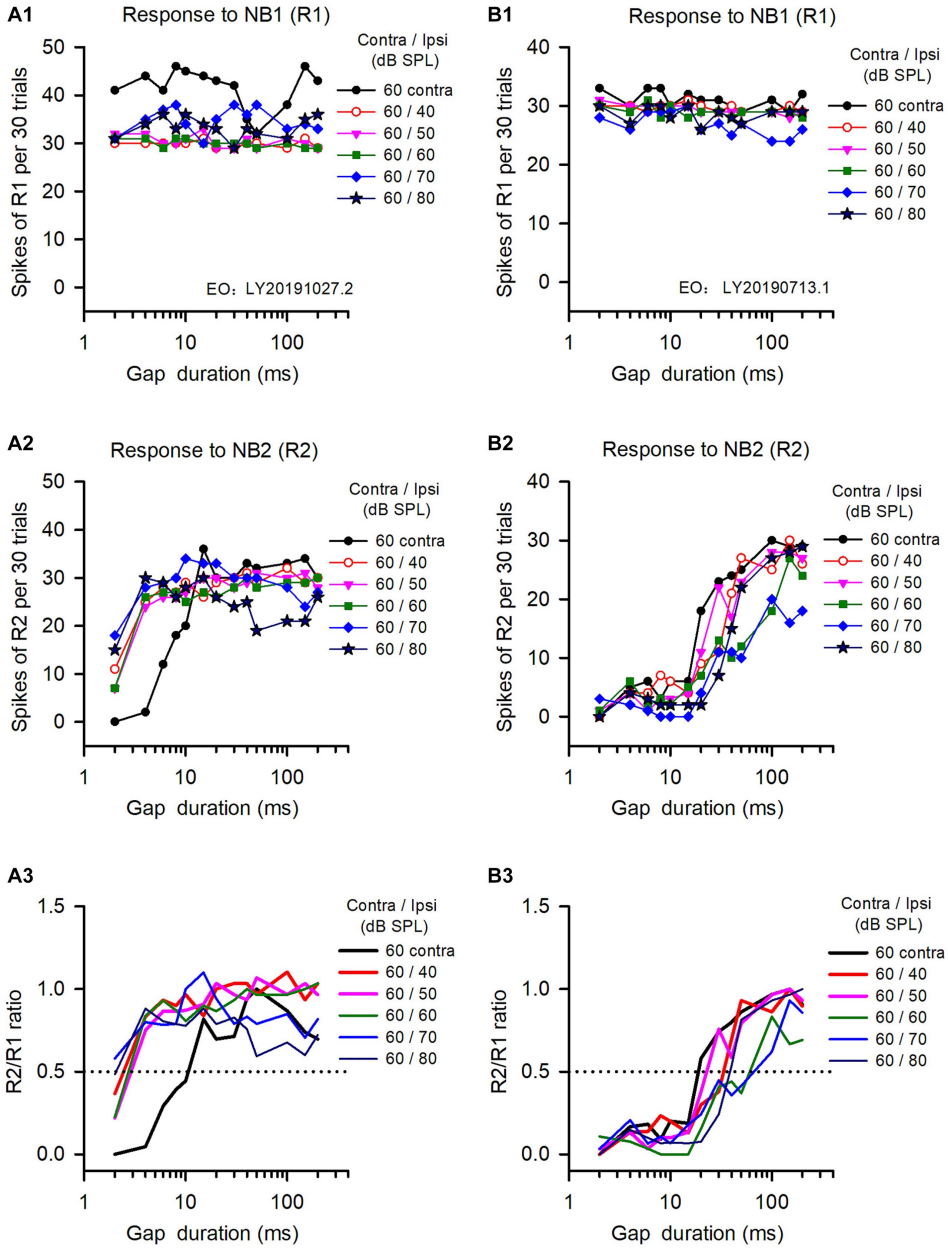
The responses of two representative IC neurons to gap stimuli determined under both monaural and binaural conditions. Panels in each column are the data from one neuron. **(A1,B1)** Responses to NB1 (R1); **(A2,B2)** Response to NB2 (R2); **(A3,B3)** R2/R1 ratio. The legend for each panel shows the noise levels presented at the contralateral (contra) and the ipsilateral (ipsi) ear. The dominant ear is the contralateral ear for both neurons. The monaural and binaural stimulus conditions (contra/ipsi dB SPL) are as follows: 60 contra (monaural, contralateral only), 60/40 (ILD +20 dB), 60/50 (ILD +10 dB), 60/60 (ILD 0 dB), 60/70 (ILD −10 dB), 60/80 (ILD −20 dB), respectively. The data determined at 0 ms gap duration are not shown due to logarithmic scale.

**Figure 3:**
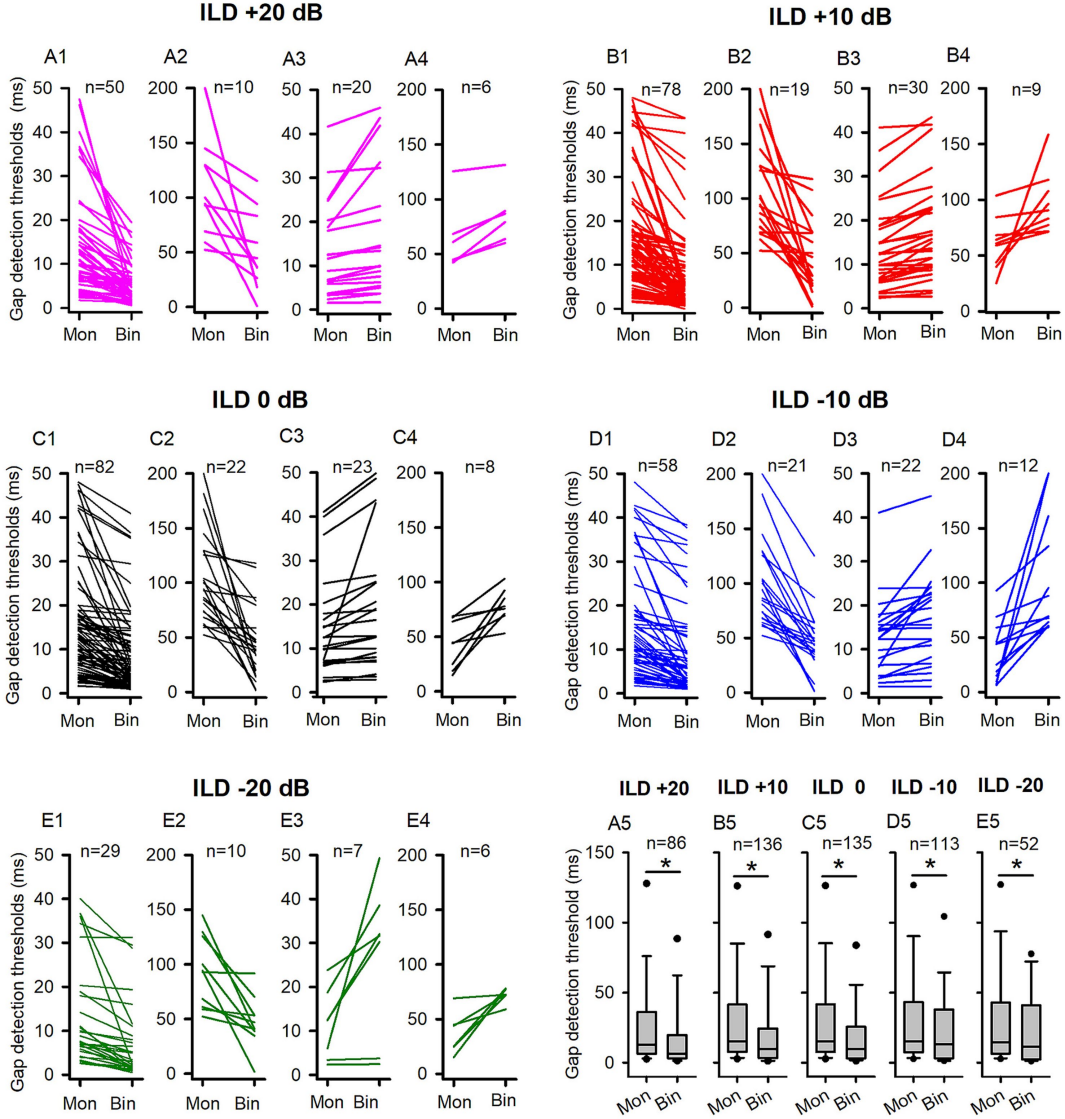
The comparison of the GDTs determined under both binaural and monaural conditions in the adult group of rats. Mon: monaural; Bin: binaural. Different ILD values indicate different binaural conditions. “n”: the number of neurons included in the data of each panel. **(A1,A2,B1,B2,C1,C2,D1,D2,E1,E2)** Each line indicates the GDT data from one neuron; for each neuron, the GDT was smaller in binaural condition than in monaural condition. Due to the wide range of the GDT values, two different scales were used on the *Y*-axis for clarity. Specifically, the data of neurons with monaural GDTs between 0 ms to 50 ms were shown in the panels **(A1,B1,C1,D1,E1)**, while the data of neurons with monaural GDTs greater than 50 ms were plotted in panels **(A2,B2,C2,D2,E2)**. **(A3,A4,B3,B4,C3,C4,D3,D4,E3,E4)** Each line indicates the GDT data from one neuron; for each neuron, the GDT was larger in the binaural condition than in the monaural condition, or equal under both conditions. Two different scales were used on the Y-axis to clearly present a wide range of GDT values. Specifically, panels **(A3,B3,C3,D3,E3)** displayed the data of neurons with binaural GDTs between 0 ms and 50 ms, while panels **(A4,B4,C4,D4,E4)** plotted the data of neurons with binaural GDTs greater than 50 ms. **(A5,B5,C5,D5,E5)** The box plots show the distributions of GDTs determined under both monaural and binaural conditions. The box plots depict the median (solid line within the boxes), quartiles (box extremities), 10th/90th percentiles (error bars), and 5th/95th percentiles (filled circles) of GDT data. * indicates a significant difference between the monaural and binaural data for comparison (*p* < 0.05, Wilcoxon Signed Rank test).

The binaural advantages in gap detection varied among the ICC neurons we analyzed, as well as the tested binaural conditions. The data in Figure 2 show the responses of two ICC neurons to gap stimuli determined under both monaural and binaural stimulus conditions. The responses to NB1 (R1) for both neurons remained stable across different gap durations (Figures 2A1,B1). In contrast, both neurons responded weakly to NB2 at short gap durations, and their responses to NB2 (R2) were gradually increased with increasing gap durations (Figures 2A2,B2); at longer gap durations, R2 recovered to the level similar to R1 (Figures 2A2,B2). Consequentially, the response ratio (R2/R1) varied with the gap durations (Figures 2A3,B3). According to the definition of neuronal GDT, the GDT of neuron A determined in the monaural hearing condition was 10.7 ms (Figure 2A3), and the GDTs of this neuron determined in binaural conditions were 2.5 ms at ILD +20 dB, 3.2 ms at ILD +10 dB, 2.9 ms at ILD 0 dB, 1.6 ms at ILD −10 dB, and 2.2 ms at ILD −20 dB, respectively (Figure 2A3). These data indicate that the GDTs were lower in the five binaural conditions than in the monaural condition, demonstrating a binaural advantage in gap detection at the tested sound stimulus conditions. For neuron B (Figures 3B1–B3), its monaural GDTs was 18.8 ms; in contrast, the binaural GDTs of this neuron were 33.5 ms at ILD +20 dB, 23.0 ms at ILD +10 dB, 71.2 ms at ILD 0 dB, 64.2 ms at ILD −10 dB, and 38.5 ms at ILD −20 dB, respectively (Figure 2B3). The GDTs of neuron B were lower in monaural condition than in the tested binaural conditions, indicating no binaural advantage in gap detection in the five tested binaural conditions (Figure 2B3).

### 3.2 Most of the ICC neurons in adult rats show binaural advantage in gap detection

For a large proportion of ICC neurons tested in the adult rats, binaural advantages were observed in gap detection under some (or all) of the five tested binaural stimulus conditions. We determined and compared the GDTs of each neuron under each binaural vs. monaural condition (Figure 3). Figures 3A1–A4 compares binaural GDTs determined under ILD +20 dB condition with the monaural GDTs. Figures 3A1,A2 show data from neurons with smaller GDTs under binaural conditions compared to monaural conditions (Figures 3A1,A2), while Figures 3A3,A4 depict data from neurons with GDTs under binaural conditions that are not less than those under monaural conditions (Figures 3A3,A4). These data show that 69.76% (60/86) of the neurons exhibit lower GDTs in the binaural hearing condition than in the monaural hearing condition, demonstrating a binaural advantage in gap detection for majority of the neurons (Figures 3A1-A4, 4A). Similarly, under each of the other four tested binaural stimulus conditions, most neurons also showed binaural advantages in gap detection: 71.32% (97/136) neurons in the ILD +10 dB condition (Figures 3B1–B4, 4A), 77.04% (104/135) neurons in the ILD 0 dB condition (Figures 3C1–C4, 4A), 69.91% (79/113) neurons in the ILD −10 dB condition (Figures 3D1–D4, 4A), and 75.00% (39/52) in the ILD −20 dB condition (Figures 3E1–E4, 4A), respectively. The Wilcoxon signed rank test has shown that the GDTs of the population ICC neurons in the adult group are significantly lower in each of the five tested binaural hearing conditions compared to the monaural hearing condition. The population data have demonstrated a significant binaural advantage in gap detection (Figures 3A5–E5, binaural vs. monaural, at ILD +20 dB, *z* = −3.890, *p* < 0.001; at ILD +10 dB, *z* = −5.296, *p* < 0.001; at ILD 0 dB, *z* = −5.9861, *p* < 0.001; at ILD −10 dB, *z* = −3.814, *p* < 0.001; at ILD −20 dB, *z* = − 2.359, *p* = 0.018).

**Figure 4:**
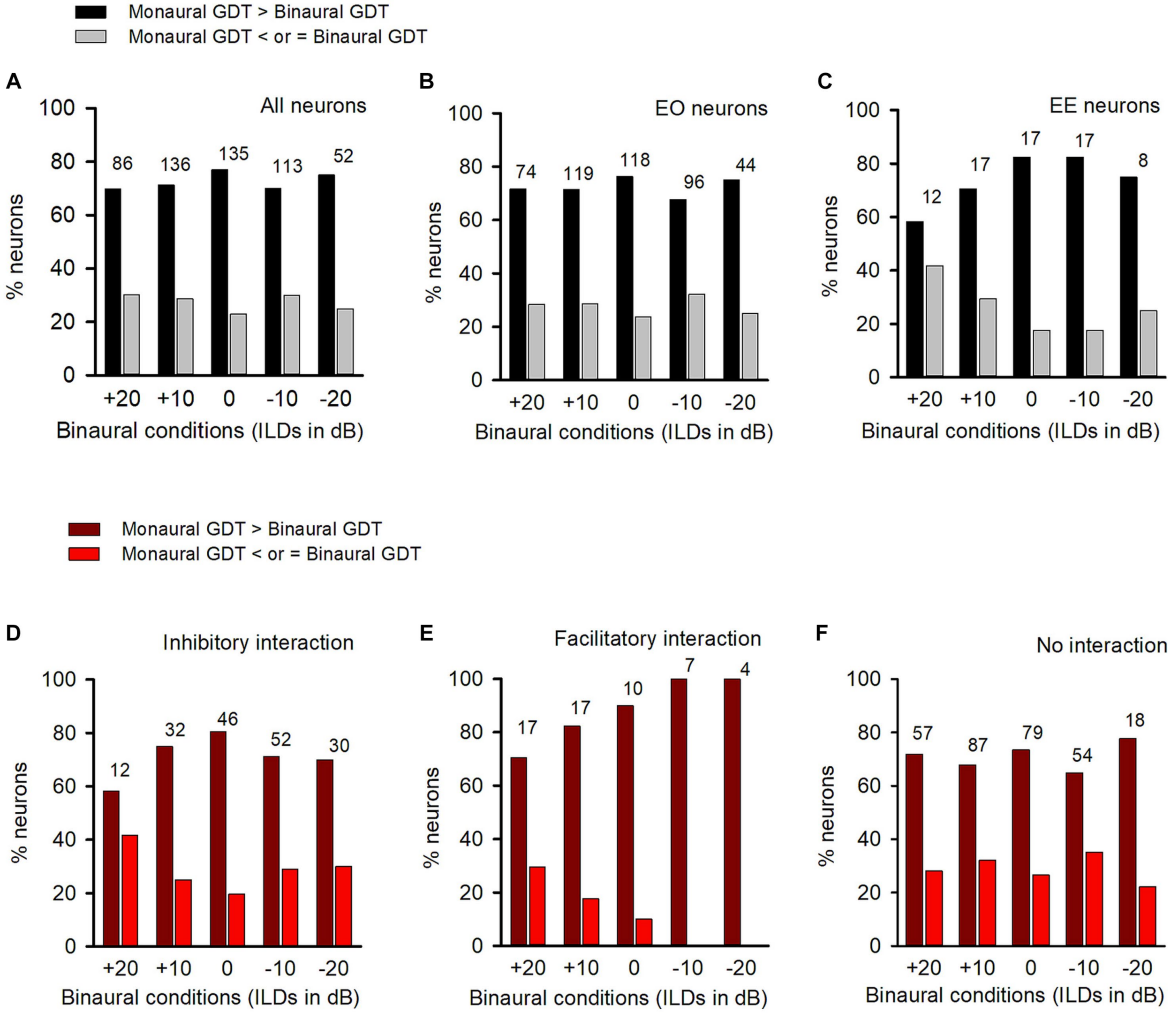
Comparison of the percentages of neurons with (or without) binaural advantages in gap detection in various categories of neurons in adult group of rats. GDT: gap detection threshold. Dark bar and dark red bar: for each neuron, GDT is larger in monaural condition than in the specified binaural condition, showing binaural advantage in gap detection; Gray bar and red bar: GDT is not larger in monaural condition than in the specified binaural conditions, showing no binaural advantage in gap detection. Each number on the top of the bars shows the total number of neurons determined in the specified binaural vs. monaural condition. **(A)** Data from all neurons; **(B)** Data from EO neurons; **(C)** Data from EE neurons. **(D–F)** Data from the neurons with inhibitory binaural interaction, facilitatory binaural interaction, and no interaction at the specified binaural vs. monaural conditions, respectively.

Among the population of ICC neurons, majority of them were categorized as EO neurons (Figure 4B), while only a small number of neurons were categorized as EE neurons (Figure 4C). Under each tested binaural vs. monaural condition, the proportion of EO neurons with a binaural advantage in gap detection was larger than the proportion of EO neurons without such an advantage (Figure 4B). Similarly, most EE neurons also showed a binaural advantage in gap detection at each tested binaural vs. monaural condition (Figure 4C).

Under each binaural stimulus condition, a neuron may exhibit inhibitory, facilitatory, or no binaural interaction based on its responses to both the binaural and monaural stimuli. We found that, in each of the binaural hearing conditions showing inhibitory binaural interaction, the percentage of ICC neurons in the adult group with a binaural advantage in gap detection was greater than those without such an advantage (Figure 4D). A similar trend was also observed for neurons with facilitatory binaural interaction (Figure 4E) or no binaural interaction (Figure 4F) under these tested binaural conditions, i.e., most of the neurons exhibited binaural advantages in gap detection under these tested binaural conditions.

### 3.3 The GDTs of ICC neurons in immature rats

To test whether the binaural advantage in gap detection observed in the ICC neurons of adult rats also exists in the ICC neurons of immature rats, we compared the GDTs of ICC neurons determined under both binaural and monaural stimulus conditions for rats in both the P14-P21 group and the P22-P30 group.

**Figure 5:**
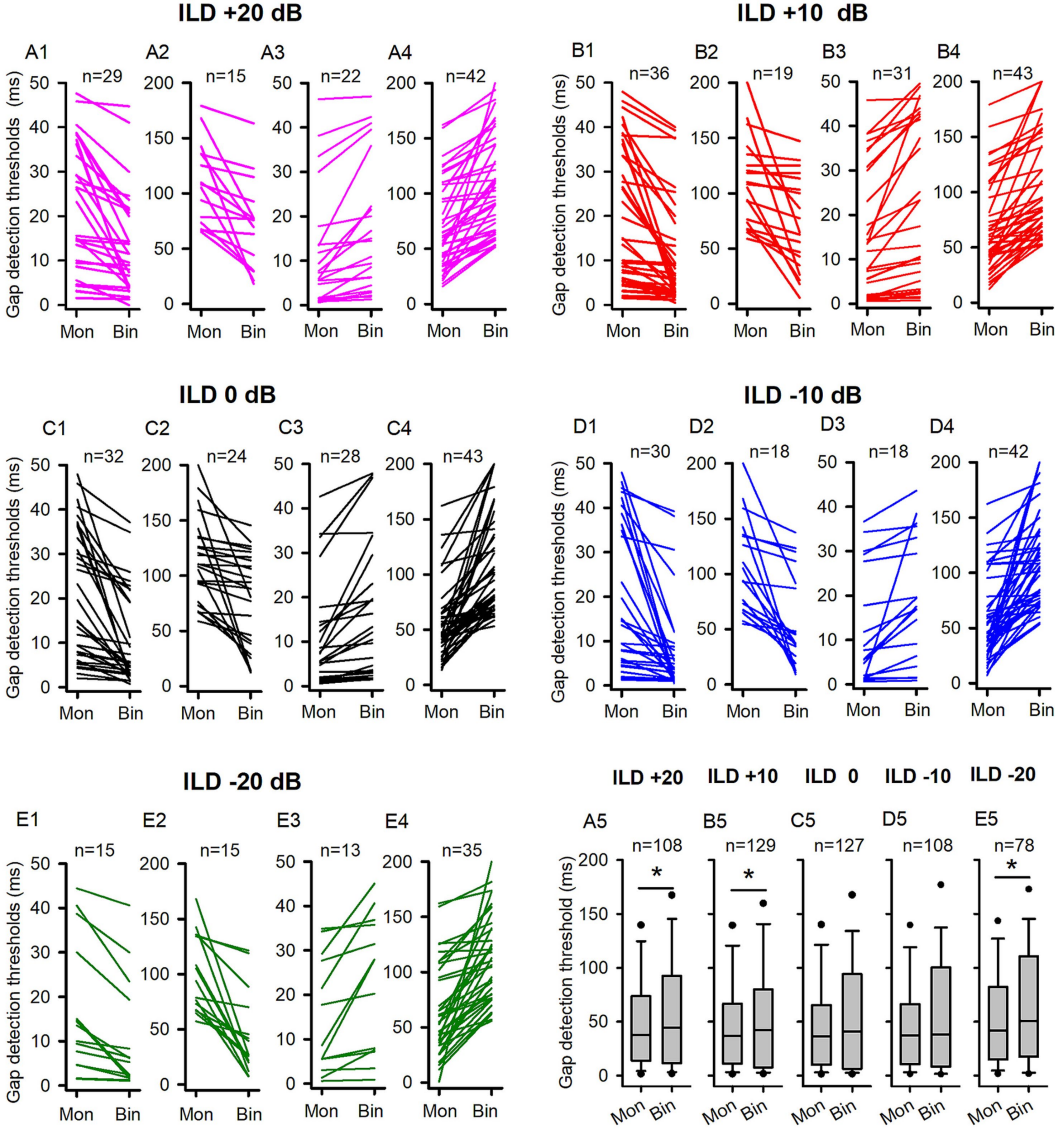
The comparison of the GDTs determined under the binaural and monaural conditions in the P14-21 group of rats. Mon: monaural; Bin: binaural. Different ILD values indicate different binaural conditions. n: number of neurons included in the data of each panel. The detail legends for each panel were the same as those shown in the figure caption of Figure 3. * indicates significant difference between the monaural and the binaural data in pairwise comparison (*p* < 0.05, Wilcoxon Signed Rank test).

The data of GDTs for all ICC neuron determined in the P14-21 group are shown in Figure 5. At ILD +20 dB binaural condition (Figures 5A1–A4, 7A1), 40.74% (44/108) neurons exhibited lower GDTs in the binaural condition than in the monaural condition. At the population neuron level, the data show a monaural advantage in gap detection at the ILD +20 dB condition (Figure 5A5, Wilcoxon Signed Ranks Test, binaural vs. monaural, *z* = −2.556, *p* = 0.011). Similarly, in the binaural conditions of ILD +10 dB (Figures 5B1–B4, 7A1) and ILD −20 dB (Figures 5E1–E4, 7A1), 42.64% (55/129) of neurons showed lower GDTs in the ILD +10 dB condition compared to the monaural condition, while 38.46% (30/78) neurons showed lower GDTs in the ILD −20 dB condition compared to the monaural condition. The population data demonstrated a monaural advantage in gap detection at ILD +10 dB and ILD −20 dB binaural conditions (Wilcoxon Signed Ranks Test, Figure 5B5, at ILD +10 dB, *z* = −2.339, *p* = 0.019; Figure 5E5, at ILD −20 dB, *z* = −2.338, *p* = 0.019). At ILD 0 dB (Figures 5C1–C4, 7A1) and ILD −10 dB (Figures 5D1–D4, 7A1) binaural stimulus conditions, 44.09% (56/127) neurons at ILD 0 dB condition and 44.44% (48/108) neurons at ILD −10 dB condition exhibited lower GDTs in the binaural conditions compared to the monaural condition, respectively; the data determined at these two binaural conditions did not show significant binaural advantage or monaural advantage in gap detection at the population neuron level (Wilcoxon Signed Ranks Test, binaural vs. monaural, Figure 5C5, at ILD 0 dB, *z* = −1.914, *p* = 0.056; Figure 5D5, at ILD −10 dB, *z* = −1.826, *p* = 0.068).

**Figure 6:**
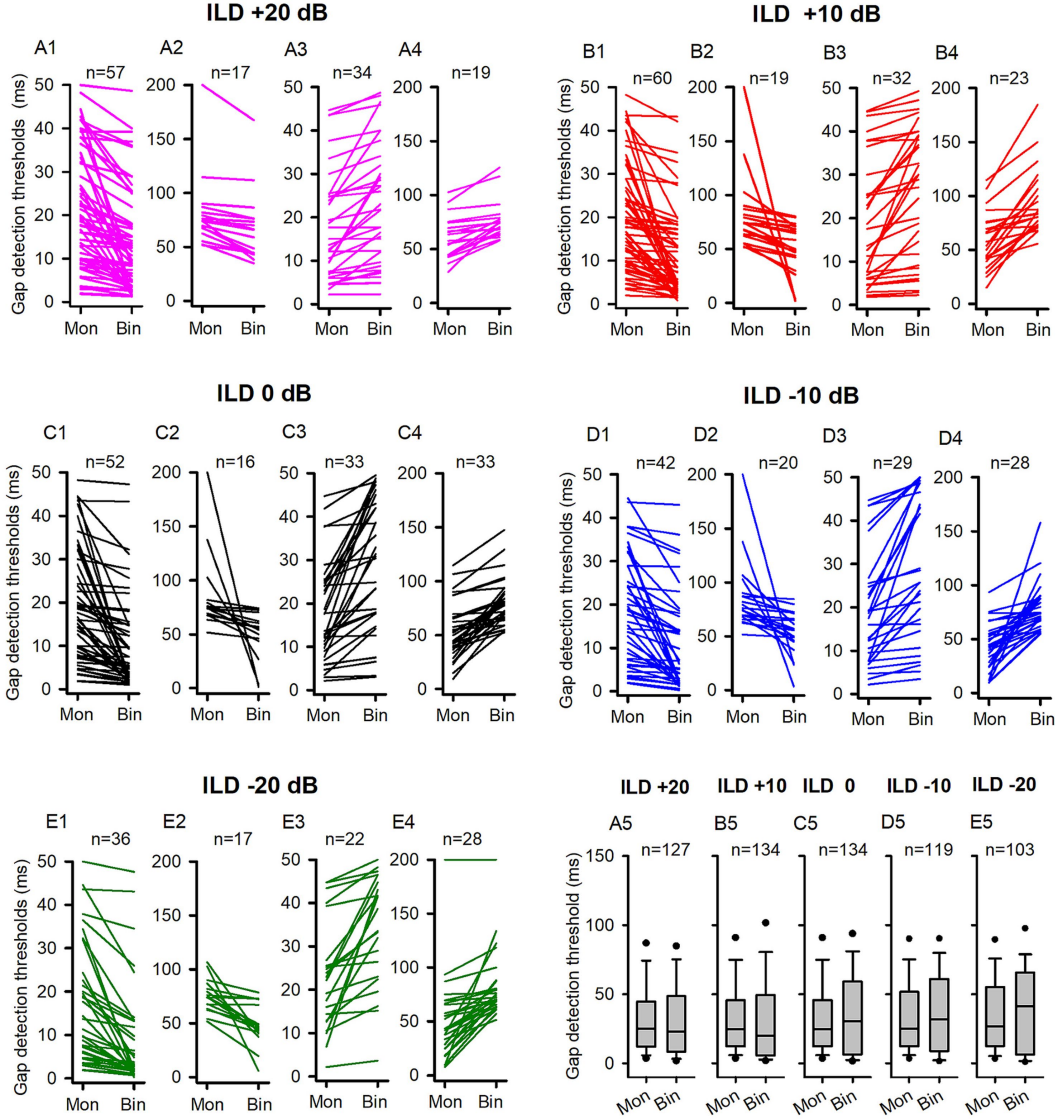
The comparison of the GDTs determined under the binaural and monaural conditions in the P22-30 group of rats. Mon: monaural; Bin: binaural. Different ILD values indicate different binaural conditions. n: the number of neurons included in the data of each panel. The detail legends for each panel were the same as those shown in the figure caption of Figure 3. No significant differences were found between the monaural and binaural data in pairwise comparison, Wilcoxon Signed Rank test, all *p* > 0.05.

In contrast to the binaural advantage in gap detection observed in the ICC neurons of adult rats, we did not observe binaural advantage in gap detection in the population ICC neurons of P14-21 rats at the five tested binaural stimulus conditions. We then investigated whether the GDTs of ICC neurons in the P22-30 group of rats exhibit a binaural advantage under each of the tested binaural vs. monaural condition. Figure 6 show the data of GDTs of all tested ICC neuron in the P22-30 group. At ILD +20 dB binaural stimulus condition (Figures 6A1–A4, 7B1), 58.27% (74/127) neurons exhibited lower GDTs at binaural condition compared to monaural condition, however, the data did not show a binaural advantage in gap detection at the population neuron level (Figure 6A5, Wilcoxon Signed Ranks Test, binaural vs. monaural, *z* = −1.708, *p* = 0.088). Similarly, we did not find binaural advantages in gap detection for the population of ICC neurons in the other four tested binaural conditions. The proportions of neurons that exhibited lower GDTs at the tested binaural conditions compared to the monaural condition were 58.96% (79/134) at ILD +10 dB (Figures 6B1–B4, 7B1), 50.75% (68/134) at ILD 0 dB (Figures 6C1–C4, 7B1), 52.10% (62/119) at ILD −10 dB (Figures 6D1–D4, 7B1), and 51.46% (53/103) at ILD −20 dB (Figures 6E1–E4, 7B1), respectively. Statistical analysis indicated no significant binaural advantage in gap detection at the population neuron level under each of the binaural vs. monaural conditions (Figures 6B5–E5, Wilcoxon Signed Ranks Test, binaural vs. monaural, at ILD +10 dB, *z* = −1.200, *p* = 0.230; at ILD 0 dB, *z* = −1.362, *p* = 0.173; at ILD −10 dB, *z* = −0.807, *p* = 0.419; at ILD −20 dB, *z* = −0.853, *p* = 0.394).

**Figure 7:**
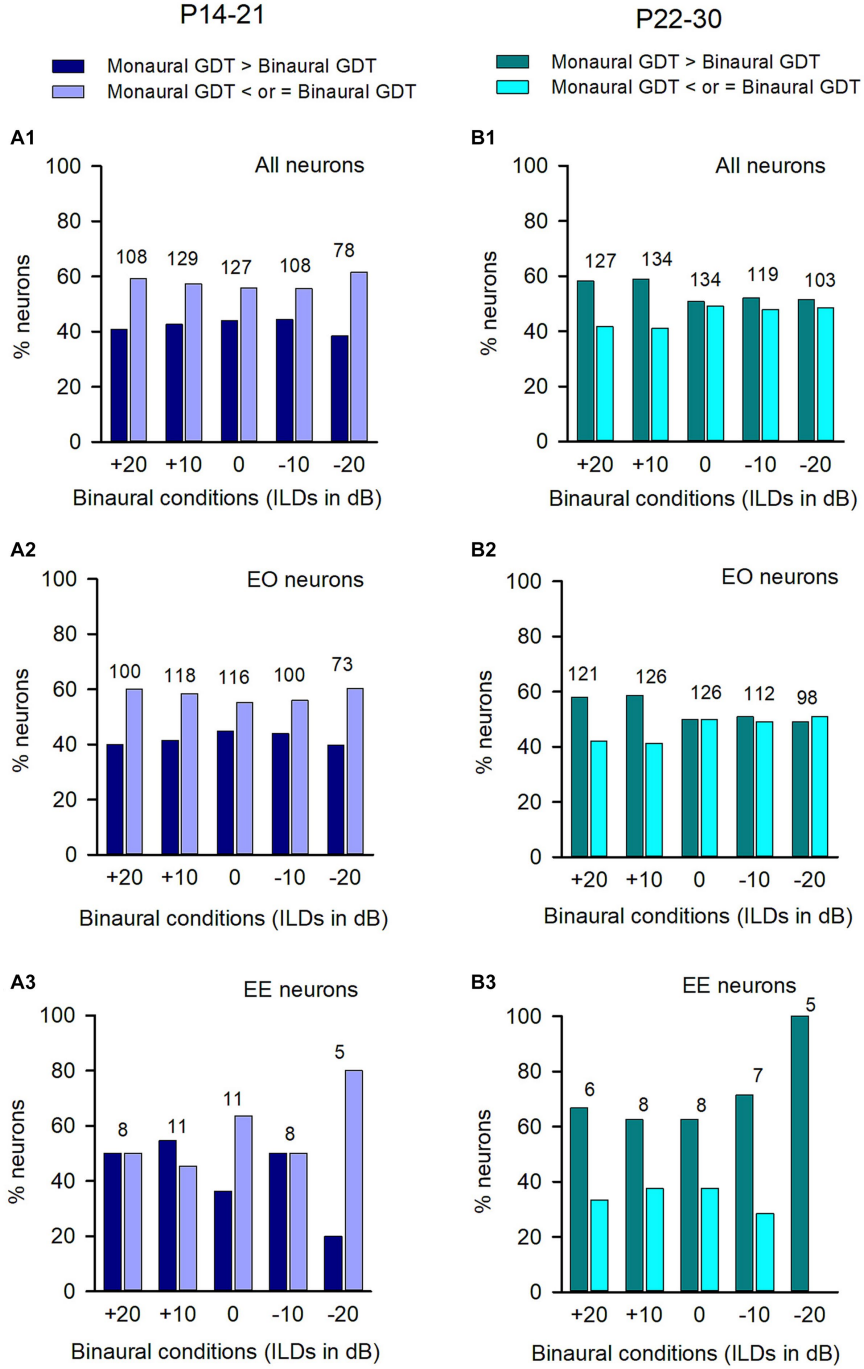
Comparison of the proportions of neurons with (or without) binaural advantages in gap detection across different categories of neurons in the P14-21 group and the P22-30 group. The left column: P14-21 group; the right column, P22-30 group. A larger GDT in monaural condition compared to the specified binaural condition indicates a binaural advantage. Each number on the top of the bars indicates the total number of neurons determined under each specified binaural vs. monaural condition. **(A1,B1)** All neurons from each group; **(A2,B2)** EO neurons from each group; **(A3,B3)** EE neurons from each group.

For the ICC neurons in the P14-21 group and the P22-30 group, the majority were EO neurons while only a small number were EE neurons (Figure 7). For both the entire population of neurons (Figure 7A1) and the EO neurons (Figure 7A2) in the P14-21 group, the proportion of neurons with binaural advantages in gap detection was smaller than those without binaural advantages in gap detection at each tested binaural vs. monaural condition; for the EE neurons in the P14-21 group, under the tested binaural hearing conditions, the proportions of neurons with a binaural advantage in gap detection were equal to (at ILD +20 dB and − 10 dB conditions), lower than (at ILD 0 dB and + 20 dB conditions), or higher than (at ILD +10 dB condition) the proportions of neurons without a binaural advantage in gap detection, respectively (Figure 7A3). For both the entire population of neurons and the EO neurons in the P22-30 group, at ILD +20 dB and ILD +10 dB conditions, the percentages of neurons with a binaural advantage in gap detection were greater than (at ILD +20 dB and ILD +10 dB conditions), or similar to (at the conditions of ILD 0 dB, ILD −10 dB, and ILD −20 dB) the percentages of neurons without binaural advantage in gap detection, respectively (Figures 7B1,B2). For the small number of EE neurons in the P22-30 group, the percentages of neurons with a binaural advantage in gap detection were greater than that without a binaural advantage in gap detection at each tested binaural vs. monaural condition in gap detection (Figure 7B3).

**Figure 8:**
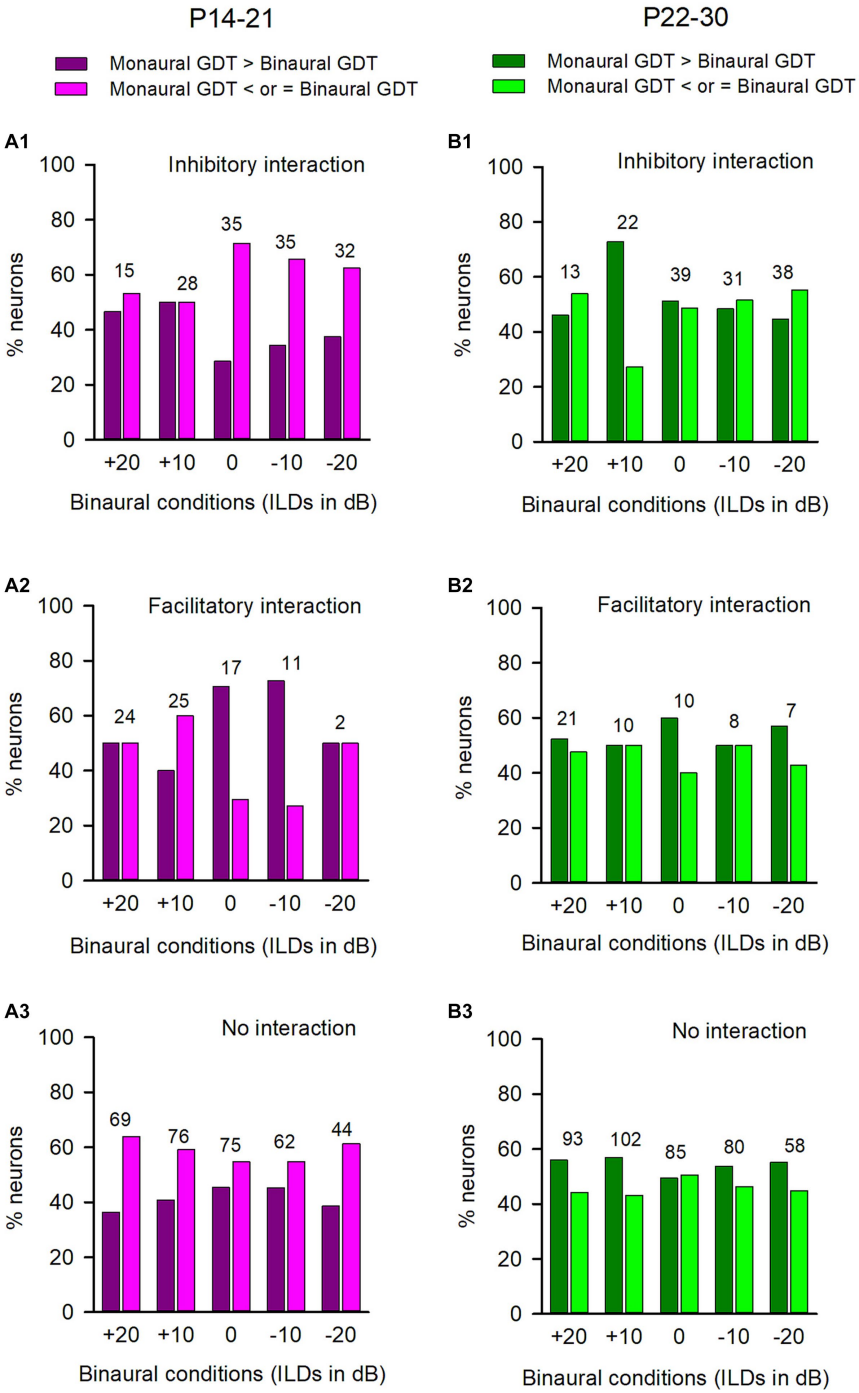
Comparison of the percentages of neurons with (or without) binaural advantages in gap detection in various binaural interaction categories in the P14-21 group and the P22-30 group. The left column: P14-21 group; the right column, P22-30 group. A larger GDT in monaural condition compared to a specified binaural condition indicates a binaural advantage in gap detection. Each number on the top of the bars represents the total number of neurons determined under the specified binaural vs. monaural condition. **(A1,B1)** Inhibitory binaural interaction; **(A2,B2)** Facilitatory binaural interaction; **(A3,B3)** No binaural interaction.

In both the P14-21 group and the P22-P30 group, we analyzed the GDTs of ICC neurons that were categorized into inhibitory binaural interaction, facilitatory binaural interaction, or no binaural interaction under each of the tested binaural vs. monaural conditions. We have demonstrated the binaural advantage in gap detection for the neurons in each of the three binaural interaction types in the adult group. However, we did not observe this binaural advantage for the GDTs of ICC neurons in both the P14-21 group and the P22-30 group. For the neurons in the P14-21 group with inhibitory binaural interaction under the tested conditions (Figure 8A1), the percentages of neurons exhibiting a binaural advantage in GDTs were smaller than the percentages of neurons without binaural advantage in GDTs at ILD 0 dB, −10 dB, and − 20 dB, respectively; however, these two percentages were similar at ILD +20 dB and ILD +10 dB. For ICC neurons in the P14-21 group with facilitatory binaural interaction at the tested conditions (Figure 8A2), the percentages of neurons with a binaural advantage in GDTs were greater than those without a binaural advantage under the ILD 0 dB and − 10 dB conditions, lower than those without a binaural advantage under the ILD +10 dB condition, or similar to those without a binaural advantage under the ILD +20 dB and − 20 dB conditions. For neurons in the P14-21 group with no significant binaural interaction under the tested conditions (Figure 8A3), the percentages of neurons with binaural advantage in GDTs were smaller than those without a binaural advantage in GDTs under all tested binaural vs. monaural conditions. For the neurons in the P22-30 group with inhibitory binaural interaction under the tested conditions (Figure 8B1), the percentages of ICC neurons with a binaural advantage in GDTs were similar to those without a binaural advantage in GDTs under ILD 0 dB and − 10 dB conditions, smaller than those without a binaural advantage in GDTs under the ILD +20 dB and ILD −20 dB conditions, or greater than those without a binaural advantage in GDTs under ILD +10 dB condition. For the ICC neurons in the P22-30 group with facilitatory binaural interaction under the tested conditions (Figure 8B2), the percentages of neurons with binaural advantage in GDTs were similar to those without a binaural advantage in GDTs at ILDs +20 dB, +10 dB, and − 10 dB, respectively; however, the percentages of neurons with binaural advantage in GDTs was greater than those without a binaural advantage in GDTs at ILD 0 dB, and − 20 dB, respectively. For the ICC neurons in the P22-30 group with no binaural interaction observed under the tested binaural conditions (Figure 8B3), the percentages of neurons with a binaural advantage in GDTs were greater than those without a binaural advantage in GDTs at the ILDs +20 dB, +10 dB, −10 dB, and − 20 dB conditions, except for the ILD 0 dB condition.

### 3.4 Age-related changes in GDTs of ICC neurons

**Figure 9:**
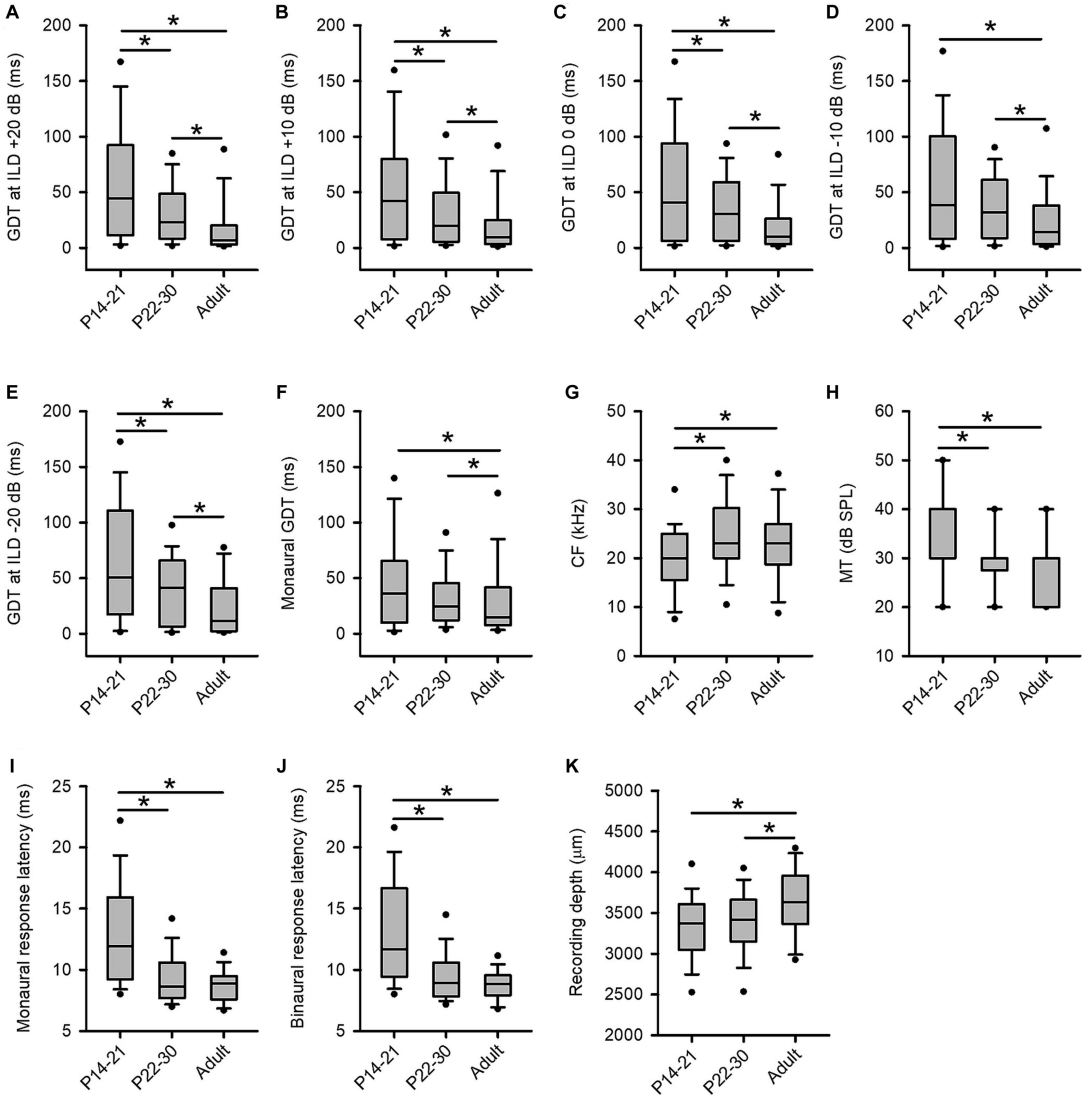
Age-related changes in GDTs and the basic properties of ICC neurons across three age groups of rats. **(A–F)** The distribution of the GDTs of ICC neurons determined under the binaural conditions (panels **A−E**) and the monaural conditions (panel **F**). **(G)** The characteristic frequency (*CF*, kHz). **(H)** The minimum threshold (MT, dB SPL). Note that the median of the MTs in the three age groups are 40 dB SPL (P14-21 group), 30 dB SPL (P22-30 group), and 30 dB SPL (adult group), respectively. The lines for the median of MTs in each group are overlapped with the box extremes. **(I)** The monaural response latencies (ms). **(J)** The binaural response latencies (ms). **(K)** The recording depth (μm).

In the present study, we observed age-related changes in GDTs of inferior collicular neurons across three age groups of rats. The binaural GDTs of ICC neurons were found to be the highest in the P14-21 group under all tested binaural conditions, except for the −10 dB ILD condition; the binaural GDTs were the lowest in the adult group under all tested binaural conditions compared to those in the other two groups (Figures 9A–E). The detailed statistical results from the Kruskal-Wallis test and Mann–Whitney test for age-related binaural GDTs are as follows: under +20 dB ILD condition (Figure 9A), df = 2, *x*^2^ = 43.887, *p* < 0.001; P14-21 group vs. P22-30 group, *z* = −3.196, *p* = 0.001; P14-21 group vs. adult group, *z* = −6.085, *p* < 0.001; P22-30 group vs. adult group, *z* = −4.652, *p* < 0.001. Under the +10 dB ILD condition (Figure 9B), df = 2, *x*^2^ = 32.879, *p* < 0.001; P14-21 group vs. P22-30 group, *z* = −2.419, *p* = 0.016; P14-21 group vs. adult group, *z* = −5.403, *p* < 0.001; P22-30 group vs. adult group, *z* = −3.791, *p* < 0.001. Under the 0 dB ILD condition (Figure 9C), df = 2, *x*^2^ = 36.837, *p* < 0.001; P14-21 group vs. P22-30 group, *z* = −2.283, *p* = 0.022; P14-21 group vs. adult group, *z* = −5.503, *p* < 0.001; P22-30 group vs. adult group, *z* = −4.481, *p* < 0.001. Under the −10 dB ILD condition (Figure 9D), df = 2, *x*^2^ = 21.193, *p* < 0.001; P14-21 group vs. P22-30 group, *z* = −1.765, *p* = 0.078; P14-21 group vs. adult group, *z* = −4.061, *p* < 0.001; P22-30 group vs. adult group, *z* = −3.565, *p* < 0.001. Under −20 dB ILD condition (Figure 9E), df = 2, *x*^2^ = 21.028, *p* < 0.001; P14-21 group vs. P22-30 group, *z* = −2.706, *p* = 0.007; P14-21 group vs. adult group, *z* = −4.318, *p* < 0.001; P22-30 group vs. adult group, *z* = −2.731, *p* = 0.006.

For the monaural GDTs of ICC neurons in the three age groups, the GDTs were the lowest in the adult group whereas the GDTs were not significantly different between the P14-21 group and the P22-30 group (Figure 9F). Kruskal-Wallis test, df = 2, *x*^2^ = 35.605, *p* < 0.001; Mann–Whitney test, P14-21 group vs. adult group, *z* = −5.632, *p* < 0.001; P22-30 group vs. adult group, *z* = −4.481, *p* < 0.001; P14-21 group vs. P22-30 group, *z* = −1.244, *p* = 0.213.

### 3.5 The basic properties of ICC neurons in the three groups of rats

We have analyzed the basic properties of three groups of ICC neurons including the distributions of the characteristic frequencies (CFs, kHz), the minimum thresholds (MTs, dB SPL), the response latencies (ms), and the recording depths (μm). We then tested the differences in CFs, MTs, response latencies, and the recording depths across the three groups of ICC neurons by Mann–Whitney Test. The CFs of the ICC neurons (range, mean ± standard deviation) in the three groups of rats are as follows: the P14-21 group, 4.0–38.0 kHz, 20.0 ± 7.3 kHz; the P22-30 group, 6.0–43.0 kHz, 24.8 ± 8.5 kHz; the adult group, 5.0–42.0 kHz, 22.5 ± 7.7 kHz. The distributions of the CFs of the ICC neurons in the three age groups (Figure 9G) show significant differences in CFs between the P14-21 group and the adult group (*z* = −2.649, *p* = 0.008), as well as between the P14-21 group and the P22-30 group (*z* = −4.304, *p* < 0.001), but not between the adult group and the P22-30 group (*z* = −1.214, *p* = 0.225). The MTs of the ICC neurons (range, mean ± standard deviation) in the three groups of rats are as follows: the P14-21 group, 20–60 dB SPL, 35.9 ± 10.4 dB SPL; the P22-30 group, 10.0–50.0 dB SPL, 29.6 ± 7.6 dB SPL; the adult group, 10.0–40.0 dB SPL, 28.4 ± 7.3 dB SPL. The distributions of the MTs across the three groups of ICC neurons (Figure 9H) exhibit significant differences in MTs between the P14-21 group and the P22-30 group (*z* = −4.996, *p* < 0.001), as well as between the P14-21 group and the adult group (*z* = −5.97, *p* < 0.001), but not between the P22-30 group and the adult group (*z* = −1.214, *p* = 0.225).

The first-spike latencies of neurons (range, mean ± standard deviation) determined at the 60 dB SPL monaural hearing condition and the 60 dB SPL binaural hearing condition in the three groups are as follows: under the monaural hearing condition, the P14-21 group, 7.51–28.21 ms, 13.11 ± 4.58 ms; the P22-30 group, 6.12–19.98 ms, 9.50 ± 2.36 ms; the adult group, 6.17–13.84 ms, 8.81 ± 1.32 ms. Under the binaural hearing condition (at 0 ILD), the P14-21 group, 7.56–26.04 ms, 12.89 ± 4.41 ms; the P22-30 group, 6.12–19.98 ms, 9.50 ± 2.36 ms; the adult group, 5.91–14.75 ms, 8.78 ± 1.46 ms. The data of monaural response latencies for the three groups of ICC neurons (Figure 9I) show significant differences between the P14-21 group and the P22-30 group (*z* = −7.345, *p* < 0.001), as well as between the P14-21 group and the adult group (*z* = −9.066, *p* < 0.001), but not between the P22-30 group and the adult group (*z* = −1.666, *p* = 0.096). Similarly, the data of binaural response latencies across the three groups of ICC neurons (Figure 9J) demonstrate significant differences between the P14-21 group and the P22-30 group (*z* = −7.557, *p* < 0.001), as well as between the P14-21 group and the adult group (*z* = −8.819, *p* < 0.001), but not between the P22-30 group and the adult group (*z* = −1.413, *p* = 0.158). In addition, the recoding depths of ICC neurons (range, mean ± standard deviation) in the three groups of rats are as follows: the P14-21 group, 1709–4,197 μm, 3,309 ± 441 μm; the P22-30 group, 2,296–4,361 μm, 3,401 ± 412 μm; the adult group, 2,300–4,503 μm, 3,611 ± 456 μm. The distributions of the recording depths for the three groups of ICC neurons (Figure 9K) exhibit significant differences between the P14-21 group and the adult group (*z* = −5.169, *p* < 0.001), as well as between the P22-30 group and the adult group (*z* = −3.853, *p* < 0.001), but not between the P14-21 group and the P22-30 group (*z* = −1.684, *p* = 0.092).

To test whether the GDTs of the recorded ICC neurons vary with their CFs, we did Pearson correlation analysis between the GDTs and the CFs of the ICC neurons. The data analysis showed that the GDTs of ICC neurons in the adult group determined in both monaural and binaural hearing conditions were not significantly correlated with the CFs of these ICC neurons (GDTs vs. *CF*, under the monaural condition, *n* = 136, *r* = −0.048, *p* = 0.579; at ILD +20 dB, *n* = 86, *r* = 0.064, *p* = 0.558; at ILD +10 dB, *n* = 136, *r* = −0.124, *p* = 0.150; at ILD 0 dB, *n* = 135, *r* = −0.057, *p* = 0.515; at ILD −10 dB, *n* = 113, *r* = −0.026, *p* = 0.782; at ILD −20 dB, *n* = 52, *r* = −0.051, *p* = 0.717). Similarly, in ICC neurons of the P22-30 group, no significant correlations were found between the GDTs and the CFs of these neurons (under the monaural condition, *n* = 134, *r* = 0.044, *p* = 0.612; at ILD +20 dB, *n* = 127, *r* = 0.164, *p* = 0.066; at ILD +10 dB, *n* = 134, *r* = 0.032, *p* = 0.714; at ILD 0 dB, *n* = 134, *r* = 0.040, *p* = 0.646; at ILD −10 dB, *n* = 119, *r* = −0.071, *p* = 0.440; at ILD −20 dB, *n* = 103, *r* = −0.099, *p* = 0.318). Moreover, the neurons in the P14-21 group showed no significant correlations between the GDTs and the CFs of these neurons under all the tested conditions (binaural, at ILD +20 dB, *n* = 108, *r* = 0.109, *p* = 0.263; at ILD +10 dB, *n* = 129, *r* = 0.157, *p* = 0.075; at ILD 0 dB, *n* = 127, *r* = 0.090, *p* = 0.312; at ILD −10 dB, *n* = 108, *r* = 0.032, *p* = 0.740; at ILD −20 dB, *n* = 78, *r* = −0.115, *p* = 0.314), except for the monaural condition (monaural, GDTs vs. CFs, *n* = 129, *r* = 0.202, *p* = 0.02).

## 4 Discussion

Previous studies on the advantages of binaural hearing have long been focused on sound localization. Humans and animals make use of the interaural difference cues to accurately perform sound localization and spatial stream segregation (Grothe et al., 2010; Middlebrooks and Waters, 2020). Abnormal binaural hearing, e.g., an acute monaural plug, degraded the directional hearing in horizontal plane (Van Wanrooij and Van Opstal, 2007; Parisa et al., 2017). The binaural advantages were also proved to be important for speech perception in a reverberate environment (Dubno et al., 2008; Yancey et al., 2021). Whereas language development (Trehub and Henderson, 1996) and speech perception in reverberation (Dreschler and Leeuw, 1990) are related to temporal resolution, little attention has been given to the function of binaural hearing on sound temporal information processing. To our knowledge, the present study is the first investigation into the binaural advantages in temporal processing at single neuron level. The major findings of the present study are as follows: The majority of ICC neurons in adult rats exhibit better neural gap detection ability in binaural hearing conditions compared to monaural hearing condition, which demonstrates binaural advantages in gap detection in the inferior colliculus; however, this binaural advantage in sound temporal information processing is not significant among the ICC neurons of the P14-30 immature rats. The results suggest a maturational process in the binaural benefit for temporal processing in the ICC.

### 4.1 The binaural advantages in speech perception and temporal processing

Previous behavioral studies have demonstrated the binaural advantages in speech perception and temporal processing. In human subjects, the binaural advantages for speech perception were observed in both normal and noisy environments (Dubno et al., 2008; Yancey et al., 2021). For subjects with normal hearing or with hearing aids, most participants showed the best performance of speech recognition in noise under binaural hearing conditions or with both ear aided (McArdle et al., 2012). For cochlear implant subjects, a bilateral advantage was demonstrated in speech recognition in a complex noisy environment (Mosnier et al., 2009). Moreover, individuals with congenital unilateral ear canal atresia show impaired speech recognition in the presence of competing speech (Siegbahn et al., 2021).

Human speech often contains distinguishing temporal features that can be used for effective auditory perception, in the present study we are trying to find if there is a binaural advantage in sound temporal information processing at neuronal level. The gap detection paradigm offers an important method to determine the temporal acuity of auditory system. Previous behavioral studies have shown that the behavioral gap detection thresholds are correlated with the ability of speech perception. Children who performed gap detection task better in infancy were subsequently reported to have larger productive vocabularies, and longer and more complex sentences than those who had performed worse in this task (Trehub and Henderson, 1996). Besides, word scores in competing babble significantly decreased with increases in gap detection thresholds (Snell et al., 2002). Furthermore, a binaural advantage in gap detection ability has been demonstrated in both human subjects and rodents (Karcz et al., 2015; Yuvaraj et al., 2023). In the present study, we attempted to investigate the neural mechanism of binaural benefit in gap detection, and our data in adult rats have provided the first evidences for binaural advantages in temporal processing at single neuron level in the central auditory system. These findings add a new function of binaural hearing at neuronal level in addition to sound localization and stream segregation, i.e., temporal processing. Since the majority of ICC neurons in adult rats demonstrate a better ability to encode sound gap duration under binaural hearing conditions than monaural hearing conditions, it is necessary and important to provide treatments for patients with unilateral hearing loss in order to restore their binaural hearing. This will improve their auditory temporal resolution and consequentially enhance speech perception and communication.

### 4.2 Binaural processing in the inferior colliculus

The inferior colliculus is an important relay station in the central auditory pathway. It receives ascending inputs from cochlear nucleus, the superior olive complex, the superior paraolivary nucleus, and the nucleus of lateral leminiscus. The superior olivary complex, the nucleus of the lateral lemniscus, and the inferior colliculus are the primary sites of binaural contact. The superior olive complex receives input from the anterior ventral cochlear nucleus from both sides. The superior paraolivary nucleus mainly project to the ipsilateral inferior colliculus (Saldana and Berrebi, 2000). The inferior colliculus receives bilateral input from both the superior olivary complex and the nucleus of the lateral lemniscus (Moore, 1991; Batra and Fitzpatrick, 2002), and it also receives descending projections from the thalamus and the auditory cortex (Winer et al., 2002). The ICC receives a large number of convergent inputs, i.e., excitatory inputs recruited by the contralateral ear and inhibitory/excitatory inputs recruited by the ipsilateral ear (Klug et al., 1999). In the present study, for each ICC neuron tested, we categorized its monaural response type and its binaural interaction type under the tested conditions. We also compared the gap detection thresholds of ICC neurons under monaural vs. binaural stimulus conditions. We found that the binaural advantage in gap detection was significant for the population of both EO neurons and EE neurons in the ICC of adult rats. Additionally, under the tested binaural conditions, majority of the ICC neurons in adult rats show binaural advantage in gap detection within each of the three categories of binaural interaction: inhibitory binaural interaction, facilitatory binaural interaction, or no binaural interaction. It appears that the binaural advantage in gap detection is prevalent in the ICC of adult rats across all binaural interaction categories. Since the ICC receives inputs from the binaural nuclei below the ICC, we should note that the binaural interaction observed in the ICC could be from superior olive complex, the nucleus of lateral leminiscus, and the inferior colliculus. Previous studies have shown that GABA(A) receptor-mediated inhibition plays a crucial role in enabling ICC neurons to process temporal information more precisely (Wu et al., 2004). In the monaural hearing conditions, the loss of inhibitory input from one ear might be a contributing factor to worse temporal processing. Previous research has shown that the peripherial hearing loss associated with the loss of motor protein prestin of cochlear out hair cells impairs the auditory temporal processing in the inferior colliculus (Walton et al., 2018). Under the monaural hearing conditions in the present study, we presented sounds only at one ear; therefore, this condition can also be considered as simulated monaural peripheral hearing loss. Consequentially, the ICC neurons should have poorer gap detection ability under monaural hearing conditions due to the loss of input from the other ear. Indeed, our data show that binaural gap detection ability is superior to monaural gap detection ability for most of the ICC neurons in adult rats.

### 4.3 Age-related changes in gap detection ability

Behavioral studies have shown that the gap detection thresholds decrease over the course of development. The gap detection thresholds are significantly worse in the human infants than in the adults (Werner et al., 1992). In the rats, the gap detection thresholds were shown to be higher in the P15 and P35 rats compared to the P64 rats (Trehub et al., 1995; Friedman et al., 2004). The neurophysiological studies have demonstrated age-related changes in auditory temporal acuity in the auditory cortex, i.e., better gap detection ability for cortical neurons in adult rats than in infant rats (Zhao et al., 2015). In the present study, for the neuronal gap detection thresholds of the ICC neurons in the three age groups of rats, we found a trend of decreasing gap detection thresholds of ICC neurons with increasing ages of rats. The results demonstrate postnatal maturation in temporal processing in ICC.

The data from the present study indicate that no significant differences in the basic properties (e.g., CFs, MTs, and the response latencies) of ICC neurons between the P22-30 group and the adult group. These data suggest that these basic properties of ICC neurons in the P22-30 rats are already mature. However, there were still significant differences in gap detection thresholds of ICC neurons between the P22-30 rats and the adult rats. It appears that the temporal acuity of ICC neurons develops to maturation slower than the basic properties of ICC neurons.

The present study demonstrates that the binaural advantage in temporal processing is significant for ICC neurons in adult rats, but not for P14-21 and P22-30 rats. These findings suggest that the development of binaural advantage in temporal processing may occur during a period from P30 to adulthood. Additionally, there is a trend indicating age-related increases in the proportions of neurons with a binaural advantage in gap detection from immature rats to adult rats (Figures 3, 5, 6). Currently, the mechanism underlying the development of binaural advantage in temporal processing remains unclear. Previous studies have shown that binaural processing undergoes postnatal refinement in the auditory cortex (Liu et al., 2021), and monaural hearing loss disrupts the development of binaural selectivity both in the auditory midbrain and cortex (Popescu and Polley, 2010). We speculate that developmental changes in binaural advantage for temporal processing are related to postnatal development and refinement of binaural processing.

### 4.4 Technical considerations

In the present study, we determined the gap detection thresholds (GDTs) of ICC neurons in rats under urethane anesthesia. Currently, there is no available single-unit data for directly comparing GDTs of ICC neurons in anesthetized and awake conditions. A recent study in mice has shown that urethane improves the responses of ICC neurons to tone stimuli (Huang et al., 2022). In this study, we measured the GDT of an ICC neuron based on its responses to two noise bursts separated by various gap durations. The GDT was calculated from a function that plotted response ratio against gap duration, where the response ratio was obtained by dividing the response to noise burst 2 by that to noise burst 1. If urethane has any effects on neuronal responses, it would influence the responses to both noise bursts; however, using a response ratio can mitigate these effects. Although potential influences of urethane on GDT values cannot be ruled out, we speculate that urethane will not significantly affect the observed trend of binaural advantage in temporal processing.

## 5 Conclusion

In summary, we found binaural advantages in gap detection ability in the ICC neurons of adult rats. This finding adds another function of binaural hearing, i.e., temporal processing, in addition to sound localization and stream segregation. However, we did not find binaural advantages in temporal processing in the ICC of both the P14-21 rats and the P22-30 rats. Furthermore, our data demonstrated age-related changes in neuronal gap detection ability in the rat ICC.

## Data availability statement

The raw data supporting the conclusions of this article will be made available by the authors, without undue reservation.

## Ethics statement

The animal study was approved by Institutional Animal Care and Use Committee (IACUC) of East China Normal University. The study was conducted in accordance with the local legislation and institutional requirements.

## Author contributions

YL: Data curation, Formal analysis, Investigation, Methodology, Validation, Visualization, Writing – original draft, Writing – review & editing. JZ: Data curation, Formal analysis, Investigation, Methodology, Validation, Visualization, Writing – original draft, Writing – review & editing, Conceptualization, Funding acquisition, Project administration, Supervision.
